# Hearing flashes and seeing beeps: Timing audiovisual events

**DOI:** 10.1371/journal.pone.0172028

**Published:** 2017-02-16

**Authors:** Manuel Vidal

**Affiliations:** Institut de Neurosciences de la Timone, UMR 7289, Aix-Marseille Université, CNRS, Marseille, France; Centre de neuroscience cognitive, FRANCE

## Abstract

Many events from daily life are audiovisual (AV). Handclaps produce both visual and acoustic signals that are transmitted in air and processed by our sensory systems at different speeds, reaching the brain multisensory integration areas at different moments. Signals must somehow be associated in time to correctly perceive synchrony. This project aims at quantifying the mutual temporal attraction between senses and characterizing the different interaction modes depending on the offset. In every trial participants saw four beep-flash pairs regularly spaced in time, followed after a variable delay by a fifth event in the test modality (auditory or visual). A large range of AV offsets was tested. The task was to judge whether the last event came before/after what was expected given the perceived rhythm, while attending only to the test modality. Flashes were perceptually shifted in time toward beeps, the attraction being stronger for lagging than leading beeps. Conversely, beeps were not shifted toward flashes, indicating a nearly total auditory capture. The subjective timing of the visual component resulting from the AV interaction could easily be forward but not backward in time, an intuitive constraint stemming from minimum visual processing delays. Finally, matching auditory and visual time-sensitivity with beeps embedded in pink noise produced very similar mutual attractions of beeps and flashes. Breaking the natural auditory preference for timing allowed vision to take over as well, showing that this preference is not hardwired.

## Introduction

We experience the outside world through external signals feeding continuously our sensory systems, and shaped by our expectations provided by prior knowledge accumulated over time from our past interactions. The brain is continuously monitoring this information to produce the most appropriate perceptual interpretation and build our phenomenal world. A crucial aspect of this process is to decide which of the external signals are to be considered as sharing the same causal origin and which are to be treated independently. In fact, most events from our daily lives are multisensory by nature, many of which audiovisual (AV): talking people, handclap, hitting hammer, etc. However, auditory and visual signals have different medium transmission latencies–light being virtually instantaneous as opposed to sound, and they are processed at different speeds–sensory afferent delays being shorter for audition than vision by about 40ms [1,2]. In fine, each of these signals reaches the brain areas responsible for integration at different moments and yet we are able to perceive simultaneity [3]. To decide for a unique bimodal event, the brain must somehow associate these two sensory signals. Three possible mechanisms have been described [2]: widening of the temporal integration window, adjustment of the offset criterion or sensory threshold.

### Temporal ventriloquism

In recent years, much attention has been given to investigate the flexibility observed in the reordering of external events in time. By analogy with spatial ventriloquism [4], temporal ventriloquism was introduced to describe the attraction of auditory and visual stimuli in the temporal domain [5]. AV disparities below 50 to 100ms were perceived as simultaneous with preceding visual stimuli being more effective in producing this illusion. The strength of this mutual attraction was measured using a Libet-like clock task [6]. The perceived timing of a flash could be forward or backward in time with a lagging or leading click (auditory capture) and to a less extent, the perceived timing of a click could be forward or backward in time with a lagging or leading flash (visual capture). Such temporal attraction was used to increase the number of flashes seen in a sequence [7]; modulate the flash-lag effect [8]; increase the temporal sensitivity to visual events [9,10]; bias the perceptual outcome in the motion-quartet illusion [11]; or modulate perisaccadic spatial mislocalization [12]. Throughout this paper, auditory and visual capture will be used to name the phenomenon that produces the temporal attraction of visual events by auditory events and vice-versa, regardless of whether such attraction is significant or not. Capture and attraction will thus be used interchangeably.

Depending on the distance to the AV source, sound will reach auditory cortex before or after the visual stimuli reaches the primary visual cortex. The “horizon of simultaneity” defines as the distance at which auditory and visual information arrive synchronously in their respective cortices [1,13], and corresponds roughly to 10m. Whether the brain adjusts the size of the temporal window of integration (TWI) according to the perceived distance remains controversial ([3,14,15] see [2] for a discussion). However, the repeated exposure to an auditory and visual stimulus onset asynchrony (SOA) indicating a shared source at a certain distance leads to perceptual adaptation. Indeed, the subjective simultaneity measured with both temporal order judgment (TOJ) and simultaneity judgment (SJ) is shifted in direction of the exposure lag [16] and this recalibration can transfer to other AV tasks [17] or to visuo-tactile and audio-tactile stimuli [18]. In these studies, the exposure to various asynchronies during the test phases could have interfered with the stored calibration [19], leading to a general underestimation of the adaptation strength. This could explain why in a recent study nearly the same recalibration could be observed from one trial to the next [20]. Interestingly, the time shifts observed after recalibration were the same for audiovisual, visuo-tactile and audio-tactile pairs of stimuli [21] suggesting that a single supramodal mechanism underlies the recalibration of multisensory time perception.

### Perceptual timing and temporal binding window

**Fig 1:**
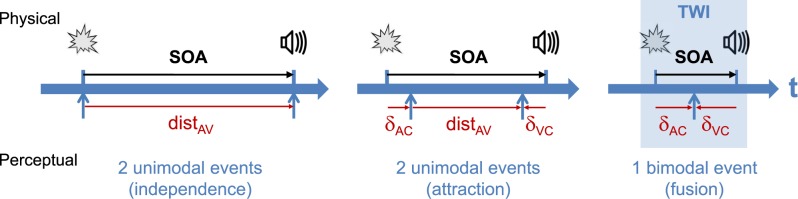
The interaction modes between discrete auditory and visual events. According to the physical temporal distance, the perceptual outcome can be either two unimodal events that are independent (perceived AV distance dist_AV_ = SOA) or attracted (auditory capture δ_AC_ or visual capture δ_VC_ not null) for large SOAs; or a fused bimodal event (dist_AV_ = 0) when closer. The temporal window of integration (TWI) can be defined as the maximal range within which visual and auditory events are totally fused (dist_AV_ = 0).

When an observer is presented with a pair of discrete auditory and visual events separated in time (SOA), the outcomes resulting from the AV interaction can be classified into three modes (illustrated in **Fig 1**). When the SOA is sufficiently large, two unimodal events are perceived, these being either independent or attracted relative to the physical offset. As auditory and visual events get closer in time, below a certain SOA only one fused bimodal event is perceived, characterizing the temporal window of integration. Typical paradigms to investigate audiovisual temporal interactions used two-alternative forced choice, either TOJ (“Which modality was perceived first”) or SJ (“Simultaneous or not”). Probability distributions as a function of SOA are then generated and fitted with psychometric (TOJ) or Gaussian curves (SJ), respectively, from which the point of subjective simultaneity is determined. Although these methodologies provided clear evidence for both attraction between senses and recalibration of simultaneity, quantitative measures of this attraction relative to an unbiased reference and the subsequent timing of perceived events are not readily available [22]. TOJ and SJ are prone to different sets of biases which produce inconsistent and uncorrelated results within a population of observers [23]. Moreover, these tasks do not quantify separately the time shift produced by auditory and visual capture, and therefore cannot really disentangle between the 3 interaction modes. Finally, the shape of the psychometric (TOJ) and Gaussian curve (SJ) does not convey the extent of the fusion zone in which only one bimodal event is perceived, and the temporal binding window is usually defined using an arbitrary threshold.

Recently, new paradigms were designed to address more precisely these timing issues and to provide quantitative measures of temporal ventriloquism. They rely on the perceived time elapsed between particular events, whether bimodal or not. In the bisection task used in [24], a probe beep-flash pair in physical synchrony was positioned within a temporal interval marked by two beep-flash pairs having the same offset (SOA). Subjects had to decide whether the probe appeared earlier or later than the interval midpoint. In the limited range reported, they found that for typical subjects the perceived midpoint shifted in time in the direction of the beep. Surprisingly, when the three bimodal pairs have their beeps and flashes perfectly synchronous, they found a huge estimation bias of the midpoint (60ms which corresponds to a second interval 35% longer than the first). Similar in principle, another task used two successive tone-delimited intervals of 1250ms containing each either the beep-flash test pair (SOA: –80, 0 and +80ms) or the synchronous beep-flash probe pair [25]. The test pair was always centered while the probe temporal position was varied within the interval. Subjects had to judge which interval contained the later stimulus. The perceived location of the AV pair always shifted toward sound of about 18ms when beeps arrived 40ms earlier in the test than in the probe interval, and of 22ms when beeps arrived 40ms later. The judgment bias when the test beep and flash were synchronous was this time only 4ms (<1%). Although bisection tasks allow relating the perceived timing to the physical timing of each event, they do not cope with the problem of rapid recalibration [19,20]. Indeed, showing only one reference interval for the forced-choice increases considerably inter-trial interactions, adding considerable noise and reducing the measured effects. This could be responsible for the strong bias reported in the no-conflict condition in [24]. Top-up trials with the adapted SOA before each test proposed in [21] is a good solution to cancel these interactions, but this study suffers from the TOJ tasks limitations for timing described earlier. Finally, a rhythmic task where subjects were presented five events and had to adjust the temporal position of the middle one to align it with the tempo defined by the other four was used in [26]. Although this paradigm is a clear improvement of bisection tasks, this study suffers from several methodological flaws, starting with the use of an adjustment task which is far less reliable than forced-choices. Further, interleaved SOA values from the large range were tested in two separate experiments with different groups of participants, which resulted in a loss of continuity in the reported time shifts. Finally, only the strength of auditory capture was measured so it could not be compared with that of visual capture to properly quantify the relative attractions.

**Fig 2:**
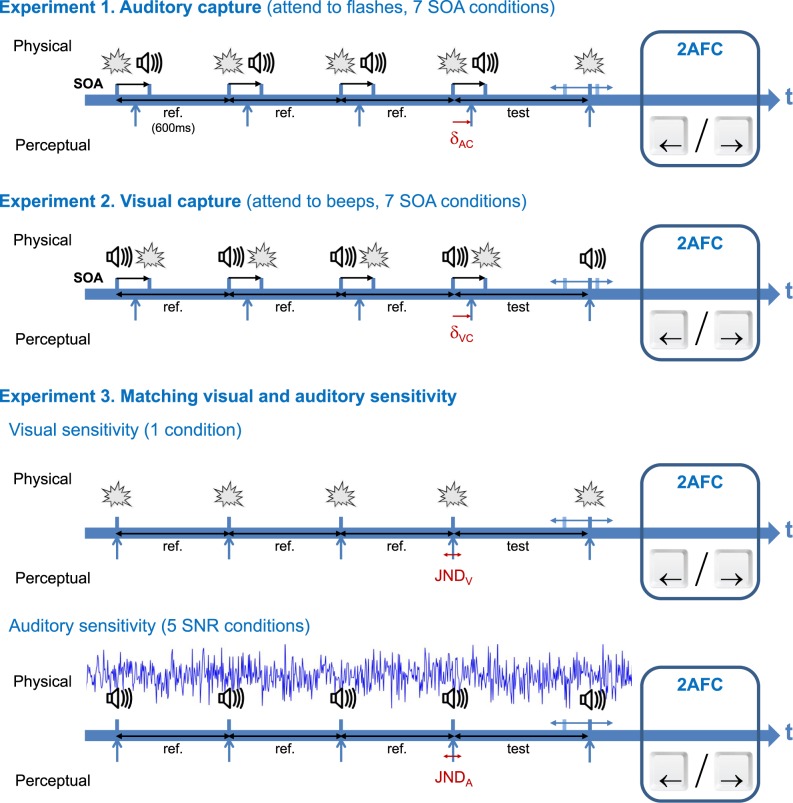
Task timelines of the experimental conditions. In **Experiment 1**, participants saw 4 beep-flash pairs regularly spaced in time defining three perceived intervals (reference of 600ms), followed after a variable duration by a 5^th^ flash (test interval). The task was to compare in a 2AFC the duration of the test interval with that of the reference intervals, while attending only to the flashes. The SOA between the flash and the beep of each pair was one of the following: –200, –80, –40, 0, +40, +80 and +200ms (positive when beep first). In the converse **Experiment 2**, participants had to attend to the beeps and the 5^th^ event was a beep (positive SOA when flash first). The auditory capture of visual flashes and the visual capture of auditory flashes produce perceptual time shifts δ_AC_ and δ_VC_ measured respectively in experiment 1 and 2. **Experiment 3** was designed to determine for each participant the signal-to-noise ratio (SNR) that impaired the auditory time-sensitivity (JND_A_) so that it nearly matched the visual time-sensitivity (JND_V_). JND_V_ and JND_A_ with beeps embedded in pink noise (5 SNR tested: no noise, +2dB, 0dB, –2dB, –4dB) were measured in separate conditions.

In order to overcome these limitations, this project used a novel paradigm inspired from the literature on auditory rhythm perception [27–29]. The time-course of each trial is illustrated in **Fig 2** (Experiments 1 & 2): four beep-flash pairs with the same offset and regularly spaced in time are presented (reference intervals), followed by a fifth unimodal event presented after a variable delay (test interval). The task is to compare the durations of the test and reference intervals in a two-alternate forced choice (2AFC). Auditory capture of flashes or visual capture of beeps is measured in trials where the fifth event is a flash or a beep, respectively. This paradigm treats temporal interactions in the most generic fashion. Since auditory and visual captures are measured separately, it allows disentangling between the three interaction modes described in **Fig 1**. This paradigm is protected from trial-to-trial interactions by presenting 4 beep-flash pairs with the same SOA before the unimodal probe. Presenting AV pairs in a rhythmic fashion also prevents known interactions between various interval durations introduced by the interleaved gap in comparison tasks [30].

### Competition between modalities

The first reported evidence of auditory dominance in the temporal domain was the notable influence of the auditory flutter rate on the perceived visual flicker rate [31]. This same effect was later used to produce the famous double flash illusion [7]. In a different context, the temporal attraction of a flash by a click was only slightly stronger than the reverse (60ms against 42ms) in the SOA range of –100ms to +100ms [6]. Furthermore, the quantitative estimations of time shifts with a bisection task confirmed that audition dominates in the perceived timing of AV stimuli [24–26]. One question that this dominance raises is whether the cue combination in the temporal domain follows the same rules as in the spatial domain, that is optimal integration in the Bayesian framework according to which the weighting of each sensory modality is proportional to its reliability [32]. The answer is not clear cut. While the temporal localization of AV stimuli was better than for either sense alone, as expected by optimal cue combination, more weight was given to audition than predicted from thresholds [24]. Finally, depending on the hosting lab, the same author could either find statistically optimal AV integration in time for continuous intervals [33] or not for intervals delimited by discrete events [25].

### Rationale

This project aimed at investigating the perceptual attraction between auditory and visual discrete events, using a novel paradigm designed to address the limitations of the TOJ, SJ and bisection tasks detailed earlier. The first goal was to measure separately the time shift produced by auditory and visual capture over a large range of audiovisual offsets in order to characterize the limits between the possible interaction modes (independence, attraction, fusion) and define quantitatively the extent of the TWI (Experiments 1 & 2). The second goal was to challenge the auditory dominance for timing predicted by the natural modality appropriateness hypothesis [34] and assess whether visual capture of beeps can be as efficient as auditory capture of flashes when both modalities have comparable reliabilities (Experiments 3 & 4).

## I. Quantifying auditory and visual temporal interactions

The temporal offset in pairs of auditory beeps and visual flashes (SOA) was manipulated in order to quantify separately the perceptual capture in the temporal dimension of visual events by auditory events (Experiment 1) and the perceptual capture of auditory events by visual events (Experiment 2). The novel method allows estimating the precise timing when the auditory and visual components resulting from the AV interaction are perceived. A large range of SOA was tested in order to observe changes in interaction modes as determined by the perceived distance between auditory and visual events (**Fig 1**). Perceptual asymmetries expected for auditory capture should be evidenced comparing the time shift of visual events attracted by preceding (SOA<0) or lagging (SOA>0) auditory events.

### Method

#### Ethics statement

For each experiment of this project, subjects gave a prior written consent after being informed of the methods used and their right to interrupt if they wished. This project was approved by the Comité d’éthique d’Aix-Marseille Université (reference 2014-12-3-06) and complies with the regulations described in the Declaration of Helsinki (2012).

#### Participants

Ten subjects (5 women and 5 men) participated in Experiment 1 (aged between 19 and 43 years, median of 24.5). Five subjects (1 woman and 4 men) from the previous group participated in Experiment 2 (aged between 19 and 43 years, median of 33). All were naïve to the purpose of the experiment except the author and all but one were right-handed.

#### Stimuli

The auditory events (beeps) were 880Hz pure tones of 20ms duration attenuated by a raised-cosine waveform (50% attenuation after 10ms) delivered through closed headphones to reduce environmental noise (BeyerDynamic DT770). The visual events (flashes) were white Gaussian blobs (σ = 0.5°) displayed for 10ms on a black background at the center of the screen (Samsung TFT running at 100Hz). A white fixation circle (0.3°) was visible throughout the trial at the same location so that the flashes were always presented at the center of the fovea. A 2-way oscilloscope was used to determine the additional lag that should be inserted between the instruction to display the flash and the instruction to play the beep so that both onsets were simultaneous when required to (precision and jitter below 1ms).

#### Procedure

Experiments took place in a dark and soundproof room, adjacent to a control room where the experimenter could monitor with an infrared camera that everything was fine. Participants sat at a table and a chin-rest maintained their eyes at 57cm from the screen center. **Fig 2** illustrates the timeline of trials for the different experiments. Trials consisted in the presentation of four beep-flash pairs (same SOA) regularly spaced in time defining three intervals (reference), followed by a fifth flash (Experiment 1) or beep (Experiment 2) defining a variable interval (test). The duration of the reference intervals was always 600ms. Subjects were instructed to attend to the flashes while disregarding the beeps (Experiment 1) or to attend to the beeps while disregarding the flashes (Experiment 2). The task was to compare in a 2AFC the duration of the test interval with the reference intervals. Answers were given by clicking the left or right mouse buttons according to whether the fifth event was perceived as occurring before (shorter test) or after (longer test) what was expected, respectively. Reaction time (RT) was limited to 2.5s in order to prevent cognitive strategies while giving enough time to answer. The SOA between the flash and the beep of each pair was one of the following: –200, –80, –40, 0, +40, +80 and +200ms (positive values when beeps came after flashes). In order to equalize learning effects across conditions, trial order was randomized by blocks of 7 with one trial for each SOA. The test interval duration was determined before each trial by an optimal Bayesian adaptive method [35] running separately for each condition. After every trial, the cumulative Gaussian best fitting the data collected until then is determined and the test value that maximizes the gain of knowledge in the next trial is computed. This fast converging method estimates in parallel the point of subjective equality (PSE) and the standard deviation (SD) of the psychometric curve with their respective theoretical 95% confidence intervals. The test durations used by the method could range from 100ms to 1100ms in steps of 10ms (see **S2 Fig** for examples of test interval distributions). Each condition was presented 60 times for a total of 420 trials. Participants could take short breaks after each block of 42 trials and were asked to take a longer middle break. At the beginning of each experiment, 21 training trials were conducted (3 per condition) to ensure that participants understood the task. The total duration of each experiment was approximately 40 minutes.

#### Data analyses

The initial pool of participants of Experiment 1 included four additional subjects that were excluded from analyses due to convergence or saturation problems with the adaptive method (see **S1 Fig** for examples of good and problematic convergence plots). For each subject and each SOA, the absolute time shift produced by the auditory capture (Experiment 1) or visual capture (Experiment 2) was computed as the difference between the last PSE estimation by the adaptive method in that condition (60 repetitions) and the reference duration (noted δ_AC_ and δ_VC_ in **Fig 2**). The last standard deviation estimation by the adaptive method, corresponding to the inverse of the psychometric slope at the PSE, was used to indicate sensitivity. The just-noticeable-difference (JND) was then computed using the linear relationship: JND = 0.6745×SD. Statistical analyses were conducted in order to tell whether the mean time shift and JND at a given SOA were different from the no-capture baseline (SOA = 0ms condition). After a one-way repeated measure ANOVA, each pair of conditions was compared following 3 steps: a normality test (Shapiro-Wilk), a variance test (Levene) and a paired Student’s t-test. The alpha value for significance was set to α = 0.00833 using Bonferroni correction for multiple comparisons on a single data set (here 6). Except otherwise stated, throughout this article all set of measures compared had distributions that did not differ from normality and had homogeneous variances. **S1 Table** reports the results of all these tests together with the effect size (Cohen’s d). Raw data files, global plots, individual plots and method convergence plots for all experiments can be found in the following public repository:

https://amubox.univ-amu.fr/index.php/s/cET4LYlOM2vLbp1

### Results

**Fig 3:**
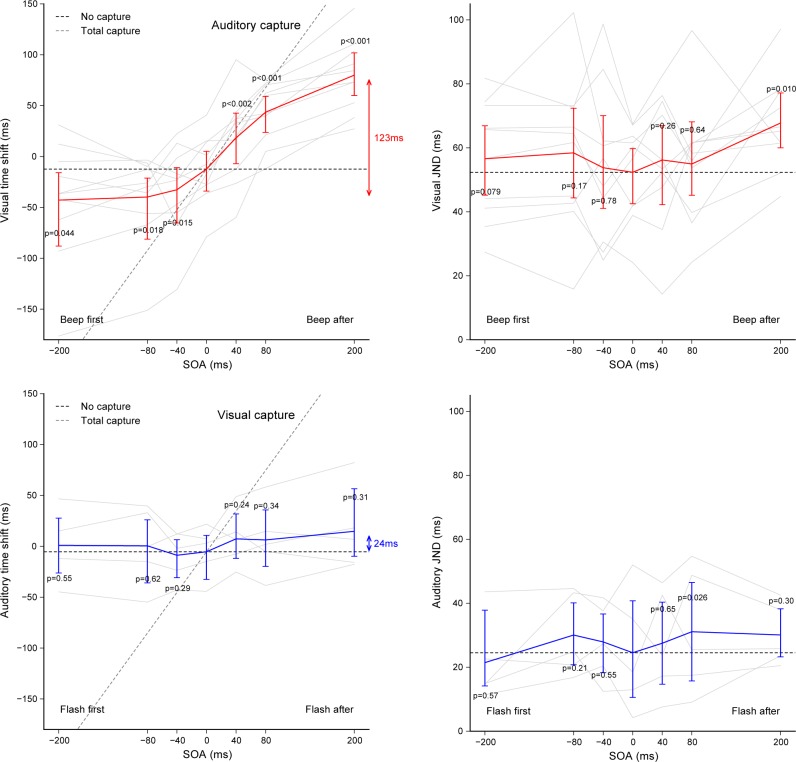
Results of Experiment 1 and 2. Visual time shift produced by auditory capture (in red, Experiment 1) and auditory time shift produced by visual capture (in blue, Experiment 2) according to the SOA **(left plots)**, and the corresponding visual and auditory JNDs **(right plots)**. The red and blue solid lines show the averages across participants (gray lines: individual results, error bars: 95% confidence intervals). Black and gray dashed lines show the predictions for absence of capture (SOA = 0ms baseline) and total capture. For each condition, p-values indicate how significant is the difference with the SOA = 0ms condition (paired Student’s t-tests with α set at 0.00833 using Bonferroni correction for multiple comparisons).

The top plots of **Fig 3**summarize the results of Experiment 1. The time shift δ_AC_ produced by the auditory capture (left) and the corresponding JND (right) are plotted as a function of SOA. Gray lines show individual results and the red line with error bars show the averages with the 95% confidence intervals estimated by bootstrap. A one-way repeated-measures ANOVA was conducted on time shifts, JNDs and RTs with the SOA condition as independent factor. There was a strong effect of SOA on time shifts (*F*(6,54) = 50.28, *p*<0.001, η_*G*_^*2*^ = 1.172): all subjects showed similar patterns and the average extent between the lowest and highest time shift was 123ms. For SOA in the range of –40 to +40ms, performance closely followed the prediction of a total capture (δ_AC_ = SOA, gray dashed line), which indicates a nearly maximal attraction of the visual flashes by the beeps. For SOA above +40ms, performance lied between total capture and the no capture baseline (δ_AC_ = δ_AC_(0), black dashed line), which shows that the attraction effect of the beeps reduced, keeping still an important time shift of 80ms for SOA = +200ms. For SOA below –40ms, the attraction reduced even faster, reaching the maximal time shift of about –40ms from the SOA = –80ms and below (normality test failed for SOA = –80ms). This difference is comforted by the higher inter-individual variability observed for SOA<–40ms than for SOA>+40ms, producing lower levels of significance. Altogether, these results indicate that the attraction when the flash occurred before the beep was more effective then when it occurred after the beep. Sensitivity indicated by the JND was not really different across SOAs (*F*(6,54) = 1.86, *p* = 0.11, η_*G*_^*2*^ = 0.063). Perfectly synchronous beeps led only to a marginally more precise estimation of the flash timing (52.3ms) when compared to the SOA = +200ms condition (67.8ms with *p* = 0.011, *d* = 1.05). Reaction times (RT) were also very similar across SOA conditions (*F*(6,54) = 0.67, *p* = 0.68, η_*G*_^*2*^ = 0.009), with an overall average of 820ms delay required to select the response and press the button.

The bottom plots of **Fig 3**summarize the results of Experiment 2. The time shift δ_VC_ produced by the visual capture (left) and the corresponding JND (right) are plotted as a function of SOA. Gray lines show individual results and the blue line with error bars show the averages with the 95% confidence intervals estimated by bootstrap. A one-way repeated-measures ANOVA was conducted on time shifts, JNDs and RTs with the SOA condition as independent factor. Time shifts were not significantly different across SOA conditions (*F*(6,24) = 1.24, *p* = 0.064, η_*G*_^*2*^ = 0.064), showing that the task irrelevant visual flashes had little effect on the perceived timing of auditory beeps. The very limited modulation of visual capture by the SOA (average time shift extent of 24ms) is in sharp contrast with that of auditory capture observed in Experiment 1 (123ms for all subjects and 104ms for the same 5 subjects, see **S3 Fig** for the results of that subgroup in all conditions). This huge difference can be quantified comparing across modalities the SOA = +80ms minus SOA = –80ms shifts: 83.4ms for auditory capture and 5.8ms for visual capture (unpaired Student’s t-test: *t*(13) = 5.37, *p* = <0.001, *d* = 2.75). Finally, the JNDs (*F*(6,24) = 0.97, *p* = 0.468, η_*G*_^*2*^ = 0.056) and RTs (*F*(6,24) = 0.84, *p* = 0.55, η_*G*_^*2*^ = 0.079) were also very similar across SOA conditions. Only the JND when the irrelevant flash came 80ms later was marginally higher compared to when it was physically synchronous (*p* = 0.026, *d* = 0.34). The overall average RT (795ms) was not different than in Experiment 1 (820ms).

### Discussion

The perception of the visual component resulting from the AV interaction could easily be forward but only little backward in time by asynchronous beeps. This auditory capture asymmetry is consistent with an early study [5] but not with [26]. In the latter, the attraction of visual flashes by auditory beeps was stronger when beeps were leading than lagging. However, the choice of an adjustment task could have introduced a systematic bias in participants’ reports, producing a global shift of the sensory attraction measured. Actually, there is an intuitive explanation to this asymmetry that is visual processing can only be compressed to a limited extent. Starting from the moment the flash hits the retina, the visual event cannot be perceived before the processing duration through the visual stream, whereas it can be retained in order to be processed together with a sound arriving later. This asymmetry could also relate to how the timing of expected stimuli in a regularly paced sequence is perceptually distorted. A recent study showed that stimuli arriving earlier are delayed whereas stimuli arriving on time or later are accelerated [36]. These changes in perceptual latencies are compatible with the conclusions of an extensive study of how recalibration transfers to other modalities [18]. Substantial auditory capture occurred after only four presentations, which is in line with fast adaptation [19,20]. In contrast, the subjective timing of the auditory component resulting from the AV interaction was not significantly shifted by asynchronous flashes. The reciprocal sensory attraction as a function of AV offset measured with this new task follows the same pattern as what previous studies reported with TOJ or SJ tasks, but with much larger effects. Namely, the time shift extent within comparable SOA range was of 120ms here against 30ms after extensive training [16,17] or after single presentations [20]. This supports the idea that presenting several times the reference interval reduces trial-to-trial interactions, which leads to larger and unbiased sensory attractions. The different set of biases occurring in each of these tasks–single stimuli interactions, order difficulty, asymmetrical decision criterion–could in fact be responsible for the large variations in attraction effect [23].

When presented in proximity, the perceived timing of visual events shifts towards the real timing of auditory events while the reverse is negligible. Previous studies provided quantitative estimations of the temporal attractions using either bimodal probes [24,25] or unimodal visual probes [26]. Since in an unbiased situation auditory beeps should be played about 40ms after visual flashes to produce perceptual synchrony, using physical synchrony as a reference to measure time shifts [24,25] might not be suitable. Only one study tried to quantify auditory capture across a large range of auditory-visual offsets [26], but the use of two separate groups of participants to measure interleaved sets of SOA points led to a surprising serrated pattern of results. Finally, measuring the converse effect–the visual capture of auditory events–was lacking in this study [26], thereby assuming a total auditory dominance in the sensory interaction. Measuring separately auditory and visual capture allowed a precise quantification of each attraction, without making any assumptions on the sensory dominance in the AV interaction and whether this interaction leads to a complete fusion–one bimodal event–or not. The perceived temporal distance between an auditory and a visual event is given by the general equation (illustrated in **Fig 1**):

$$dist_{\text{AV}}\left(SOA\right)=SOA-\delta_{\text{AC}}\left(SOA\right)+\delta_{\text{VC}}\left(SOA\right)$$ (1)

**Fig 4:**
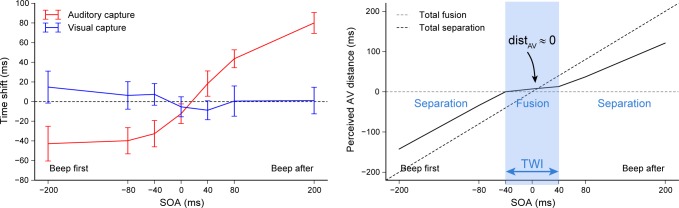
The perceived AV distance. Time shifts produced by auditory capture δ_AC_ and visual capture δ_VC_ with the same SOA sign convention, averaged across participants **(left plot)** and the resulting estimation of the auditory-visual distance dist_AV_ **(right plot)**. Error bars indicate inter-individual standard errors. The black and gray dashed lines on the bottom plot show the predictions for total fusion (dist_AV_ = 0) or for total separation (dist_AV_ = SOA), which are used to distinguish 3 areas of AV interaction along the SOA axis: separation, fusion and separation. The temporal window of integration (TWI) corresponds roughly to the [–40ms, +40ms] range.

A distance close to zero corresponds to the simultaneous perception of the auditory and visual events. Determining this distance for a large range of SOAs allows describing properly the changes between interaction modes (independence, attraction and fusion). The auditory capture (δ_AC_) and visual capture (δ_VC_) measured respectively by Experiment 1 and 2 are shown in **Fig 4**(top plot) with the same SOA sign convention (beep first / beep after). Based on the data collected and the equation above, the average auditory-to-visual perceived distance as a function of SOA was estimated (bottom plot of **Fig 4**). For SOAs in the range of –40ms to +40ms, the perceived auditory-to-visual distance is null indicating that only one AV fused event is perceived, which provides a quantitative description of the temporal window of integration. For SOAs smaller than –40ms and greater than +40ms, distance grows with the SOA showing that two separate auditory and visual events are perceived. Curiously, even for –200ms and +200ms the distance was not enough to reach total independence (black dashed-line asymptote), and interaction remained in the attracted mode. Since the duration of reference intervals was 600ms, larger SOA values cannot be tested using this methodology as interactions could happen with either neighboring event.

The novel paradigm developed here allowed describing accurately the different auditory and visual temporal interaction modes according to the offset, without any of the limitations found in the literature. First, the auditory capture of visual flashes as well as the visual capture of auditory beeps where quantified in separate conditions, which enabled computing the perceived auditory-visual distance without making the assumption of an auditory dominance. Second, a wide range of SOA was tested, which provides a more complete description of how auditory and visual capture change with temporal distance. Third, using four beep-flash pairs with the same offset at the beginning of each trial prevented trial-to-trial interactions related to fast recalibration [19], which contrasts with single pair judgments, bisection tasks or interval comparisons. In conclusion, even though the visual capture observed was very limited, it was complementary to the auditory capture so that a fusion zone could be observed (also providing a sanity check). In natural conditions in which auditory and visual saliency are standard, auditory capture shifts the perceived timing of flashes towards beeps much more than visual capture shifts the beeps towards flashes. Within the temporal window of integration where AV fusion occurs, it is the combination of both the flash and the beep temporal shifts that cancels perceptually the physical distance between the two unimodal events.

Finally, the temporal sensitivity observed for beeps (JND of 24.5ms, Experiment 2) was notably better than for flashes (JND of 52.3ms, Experiment 1). Could differences in reliability reflected by the auditory and visual sensitivities in natural conditions be responsible for the strong disparity between auditory and visual capture?

## II. Auditory and visual capture with equal reliability

The strong auditory influence on perceived visual timing can be explained by the modality appropriateness hypothesis according to which vision would be specialized for spatial processing and audition for temporal processing [34]. One can wonder whether this specificity is hardwired or not, that is, if the brain has selectively evolved to rely more on the auditory modality when processing temporal features of sensory inputs. Another alternative would be that when two modalities interact, the temporal integration adapts to the reliability of each modality in the given context, which in most situations but not necessarily would favor audition. As reviewed in the introduction, it is still controversial whether AV temporal integration is statistically optimal. Some found a clear auditory dominance going beyond predicted from its reliability [24], regardless of the attentional instruction to ignore audition or the deterioration of auditory signal [37], while others found the weighting of each modality in the integration to be compatible with the observed sensory reliability [33]. Finally, the strong auditory interference on the capacity to memorize a visual sequence [38] led to the conclusion that temporal encoding of visual sequences relies on the auditory cortex, favoring the hardwired hypothesis. The following experiments were designed to tackle this problem with a different approach: once visual and auditory temporal sensitivities are matched, will visual events capture auditory events with the same strength as auditory events capture visual events?

In Experiment 3, temporal sensitivity to auditory events was impaired by embedding the beeps in pink noise (sound of a waterfall) with various signal-to-noise ratios (SNR). For each participant, the SNR for which auditory and visual sensitivities are comparable will be estimated and used in Experiment 4 to measure how strong auditory and visual events attract each other when given equal reliabilities.

### Method

#### Participants

Twelve subjects (7 women and 5 men) participated in Experiment 3 (aged between 17 and 38 years, median of 21.5). Nine subjects (5 women and 4 men) from the previous group participated in Experiment 4 (aged between 17 and 38 years, median of 25). All were naïve to the purpose of the experiments except the author and all were right-handed.

#### Procedure

In Experiment 3, the task was similar to the previous (see **Fig 2**) except that now the events in the reference and test intervals were unimodal: either visual flashes (1 condition) or auditory beeps (5 conditions). In auditory conditions, a continuous background pink noise was added to the beep sequence. Noise started and ended 300ms before the first and after the last beep, and for greater acoustic comfort, its intensity was linearly ramped for 150ms (fade-in and fade-out). One of the following 5 SNR between beeps and pink noise were tested: +∞ (no noise), +2dB, 0dB, –2dB or –4dB (beep signal intensity was varied while pink noise intensity was constant). The task was again to compare in a 2AFC the test interval duration with the reference intervals, and participants’ answers were given by clicking the left/right mouse buttons. Each condition was repeated 80 times instead of 60 as previously in order to improve the adaptive method’s final estimation of the standard deviation of the cumulative Gaussian best fitting the psychometric curve [35]. The experiment contained 480 trials, with breaks between each block of 48 trials and lasted approximately one hour including the training phase.

In Experiment 4, the same method as in Experiment 1 and 2 was used to measure the auditory and visual capture within participants in a single session. Only 3 SOA conditions were kept: –80ms, 0ms and +80ms. These values proved to show a clear attraction effects in the previous experiments. Pink noise was delivered in all conditions, using for each subject the SNR level that matched auditory and visual JNDs as determined in Experiment 3. Each condition was repeated 60 times and auditory and visual captures were assessed in separate blocks of 36 trials (12 repetitions of each SOA). Blocks alternated between trials measuring auditory or visual capture, the instruction being given at the beginning of each block accordingly (attend to flashes or to beeps). The experiment contained 360 trials with breaks between each block and lasted approximately 45 minutes including the training phase.

#### Data analyses

Three subjects were removed from the initial pool of participants of Experiment 3 and three more from Experiment 4 due to convergence problems with the Bayesian adaptive method. For each condition, time shifts and JNDs were extracted from the last trial mean and standard deviation estimations by the adaptive method. In Experiment 4, statistical analyses were conducted to compare the mean time shift and JND for the SOA = –80ms and SOA = +80ms conditions with the no-capture baseline (SOA = 0ms). After a two-way repeated measure ANOVA (auditory and visual capture is now a between-subject factor), each pair of conditions was compared as previously. The alpha value for significance was now set to α = 0.025 correcting for the two comparisons on each data set. **S2 Table** reports the results of all these tests together with the effect size (Cohen’s d).

### Results

**Fig 5:**
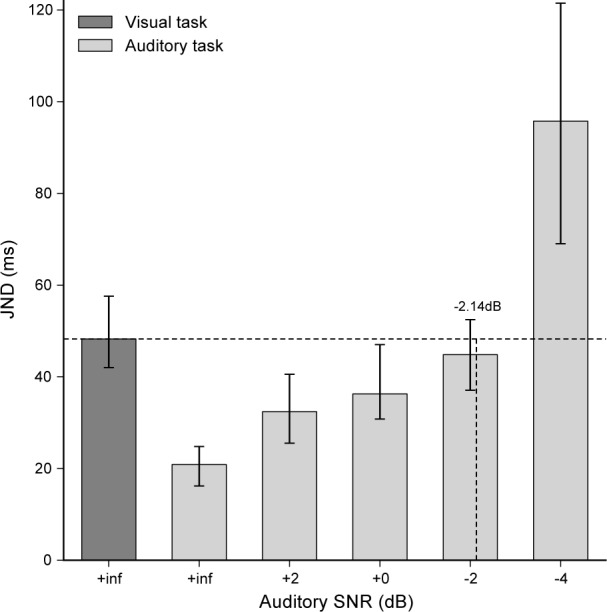
Results of Experiment 3. Average visual sensitivity JND_V_ (dark gray bar) and auditory sensitivities JND_A_ according to the signal-to-noise ratio (light gray bars). Error bars indicate the 95% confidence intervals. For each subject, the SNR that raises JND_A_ to the level of JND_V_ was determined using linear interpolation between the first successive SNR pair for which the JND_A_ segment contained JND_V_ (horizontal dashed line). As an example, the group SNR of –2.14dB was found in the [–2dB, –4dB] segment.

The average visual JND_V_ and auditory JND_A_ for each SNR measured by Experiment 3 are shown in **Fig 5**. Increasing the SNR in noise conditions reduced auditory time-sensitivity as shown by the increasing JND, while it had virtually no effect on the PSE (see **S4 Fig**). RTs were also very similar across conditions. For each subject, the SNR that raises auditory JND_A_ to the level of visual JND_V_ was determined using linear interpolation between the first two successive SNR values for which the auditory JND_A_ segment contained the visual JND_V_. This occurred for 2 subjects in [+2dB, 0dB]; for 5 subjects in [0dB, –2dB]; for 3 subjects in [–2dB, –4dB]. For the remaining 2 subjects, since JND_A_ with a SNR of –4dB was still lower than JND_V_, the matching SNR was arbitrarily set to the maximal level of –4dB.

**Fig 6:**
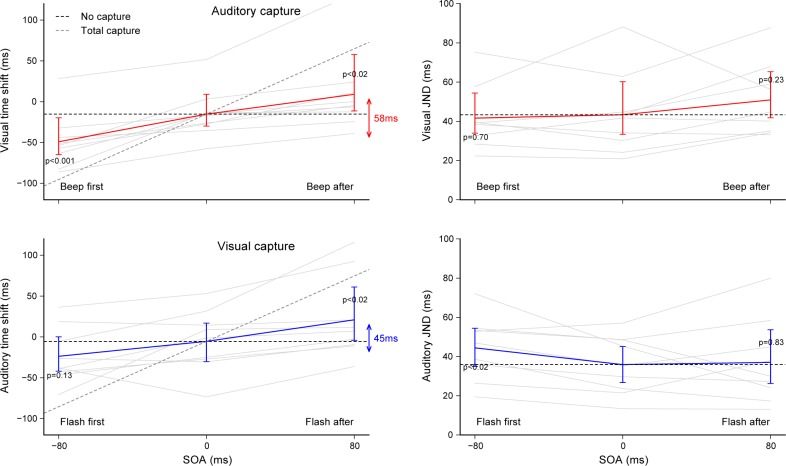
Results of Experiment 4. Time shifts produced by auditory and visual capture according to the SOA **(left plots)** and the corresponding visual and auditory JNDs **(right plots)**. The red and blue lines show the averages across participants (gray lines: individual results, error bars: 95% confidence intervals). Black and gray dashed lines show the predictions for absence of capture (SOA = 0ms baseline) and total capture. For each condition, p-values indicate how significant is the difference with the SOA = 0ms condition (paired Student’s t-tests with α set at 0.025 using Bonferroni correction for multiple comparisons).

**Fig 6** summarizes the results of Experiment 4. Time shifts produced by the auditory capture and visual capture are plotted as a function of SOA (left plots). A two-way repeated-measures ANOVA was conducted on time shifts and JNDs with capture modality and SOA as independent factors. SOA had a significant effect on time shifts (main effect: *F*(2,16) = 34.80, *p*<0.001, η_*G*_^*2*^ = 0.308) but not the capture modality (main effect: *F*(1,8) = 0.66, *p* = 0.44, η_*G*_^*2*^ = 0.043). When attending to visual flashes, time shift increased by 24.3ms (*p*<0.015, *d* = 0.59) and decreased by 33.8ms (*p*<0.001, *d* = 1.06) for a 80ms lagging and leading beep, respectively. When attending to auditory beeps, time shift increased by 26.4ms (*p*<0.015, *d* = 0.59) and marginally decreased by 18.3ms (*p* = 0.137, *d* = 0.51) for a 80ms lagging and leading flash, respectively. The decrease was not statistically significant because of a deviant subject in this condition (the lowest gray line). The average amplitude between the lowest and highest time shift was not significantly different between auditory capture (58.1ms) and visual capture (44.7ms), and each of these were significantly different from 0 (t-test against single value: *p*<0.001, *d* = 2.20 and *p*<0.01, *d* = 1.15, respectively). For both auditory and visual modalities, performance lied clearly in between total capture (gray dashed line) and the no capture baseline (black dashed line). Taken together, when temporal sensitivities match, the effect of beeps on the visual flash timing was not really different than the effect of flashes on the auditory beep timing. Visual and auditory JNDs corresponding to the auditory capture and visual capture are plotted as a function of SOA (right plots of **Fig 6**). There was no noticeable effect of the SOA on JNDs except for the visual capture with an SOA of –80ms. The average JND_V_ (45.3ms) was slightly higher than the average JND_A_ (39.1ms), but this difference was not significant (capture modality main effect: *F*(1,8) = 1.94, *p* = 0.20, η_*G*_^*2*^ = 0.033). In Experiment 3, visual sensitivity was measured without noise, as the desired SNR was not yet determined. The noise added in Experiment 4 could have interacted with visual temporal judgments, but this was not the case. For the group of 9 subjects kept in Experiment 4, visual sensitivity was preserved (for SOA = 0ms, the JND_V_ was 43.6ms without noise and 43.4ms with noise) whereas auditory sensitivity was expectedly impaired (JND_A_ increased from 20.1ms to 35.9ms with noise).

**Fig 7:**
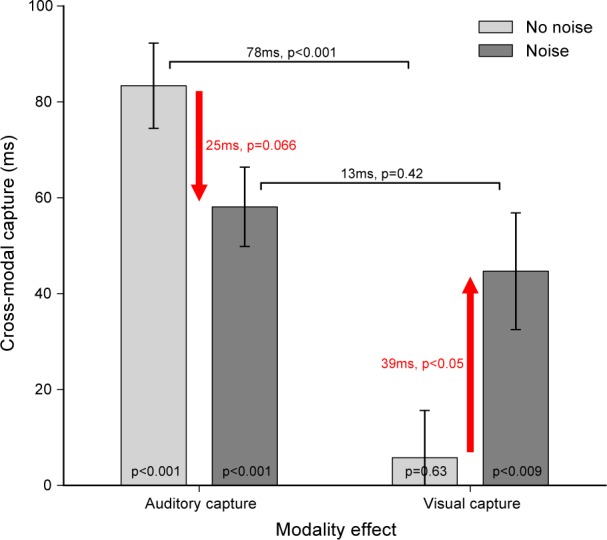
Effect of noise on the sensory attraction. Cross-modal capture (difference in time shift between +80ms and –80ms SOA) according to the capture modality (auditory or visual) and noise condition. *No noise* conditions were measured in Experiments 1 and 2 (light gray bars) while *Noise* conditions were measured in Experiment 4 (dark gray bars). Statistical comparisons were done with unrelated Student’s t-test except for the within comparison of visual and auditory capture with noise (Experiment 4).

In order to compare these results with the auditory and visual capture obtained without noise (data collected in Experiment 1 and 2), the cross-modal capture–defined as the difference in time shift between +80ms and –80ms SOA conditions–was computed for each capture modality and noise condition (see **Fig 7**). Adding noise tended to reduce the auditory cross-modal capture (–25ms, *t*(17) = 1.97, *p* = 0.066, *d* = 0.90), produced a significant visual cross-modal capture (+39ms, *t*(12) = 2.29, *p*<0.05, *d* = 1.12) and cancelled the significant difference between auditory and visual cross-modal capture (from 78ms without noise, *t*(13) = 5.37, *p*<0.001, *d* = 2.75, to 13ms with noise, *t*(8) = 0.86, *p* = 0.42, *d* = 0.41).

### Discussion

The signal-to-noise ratio between the beep sequences and a background pink noise that lowered auditory temporal sensitivity to that of vision was determined individually in a unimodal context. With this level of noise during the audiovisual stimulations, the effect of beeps on visual timing was comparable to the effect of flashes on auditory timing. Both auditory and visual captures lied roughly in the middle between total capture and the no capture baseline. This demonstrates that when auditory and visual sensitivities are comparable, visual capture becomes possible, that is, task irrelevant visual events do produce noticeable effects on auditory temporal judgments rather similar in strength to the converse auditory capture. Although not statistically significant, according to the Bayesian framework this slight difference could stem from an underestimation of the noise required to provide equal capabilities to auditory and visual temporal judgments. Indeed, for two subjects the SNR was arbitrarily set to –4dB–the maximal level of noise tested–leaving auditory time sensitivity with noise still better than visual sensitivity. These two subjects showed a stronger auditory capture than visual. Finally, auditory and visual sensitivities were matched in a unimodal context. In the bimodal context where auditory and visual captures were determined, the noise added to impair auditory temporal judgments could have also affected visual judgments but this was not the case. While auditory time-sensitivity was expectedly reduced, visual time-sensitivity remained at the same level as without noise.

In natural conditions in which auditory and visual saliency are regular, the auditory capture that shifts the perceived timing of visual flashes towards beeps is much stronger than the reverse visual capture of beeps. Degrading the auditory temporal sensitivity with a background pink noise reduced the time shift produced by the auditory capture and created a notable visual capture otherwise insignificant. Therefore impairing auditory sensitivity with noise had consequences on both visual capture–stronger than without noise, and auditory capture–weaker than without noise. Lowering auditory reliability to break the natural modality appropriateness [34] by giving the same temporal sensitivity to vision and audition resulted in equivalent cross-modal capture. This finding supports the idea of a sensory weighting in the temporal integration based on the reliability of each modality [33] just like in the spatial domain [32,39,40]. It rejects the idea that duration perception is mandatorily linked to auditory processing under most circumstances [37] or that auditory dominance goes beyond what predicted from its reliability [24]. The task used in the first study [37] was a forced-choice where participants had to judge whether an interval delimited by unimodal (A or V) or AV bimodal events was short or long given previously delivered short (200ms) and long (800ms) reference intervals. The relative sensory intensity was varied but the outcome was similar as soon as auditory events were delivered together with the visual events: the perceived mid-point interval durations were notably underestimated compared to unimodal visually-defined intervals by about 150ms. The main concerns with this study come from the task that involves a memorization of two reference durations. First, these references were given unimodally and it was hard to control which modality was actually used for the comparisons. It might change from trial to trial but also across participants. Second, it is impossible to disentangle between an audiovisual interaction occurring at the memorization level, at the decision level or at the perceptual level. In the present work, the reference interval was always 600ms which lies within the same range. In the unimodal conditions (Experiment 3) there was barely any difference between the visual PSE (578ms) and the auditory PSEs whichever the noise level (between 586ms and 607ms, see **S4 Fig**). This shows that with a task in which the memorization load is reduced by presenting several times the reference interval before testing directly the perceived durations, there is no effect of the modality on timing accuracy. Auditory dominance was also reported for the memorization of rhythmic sequences, using another audiovisual type of interaction [38]. The temporal encoding of visual sequences of gabor phase alternations was significantly impaired by irrelevant beep sequences, while the interference of incongruent visual information with auditory rhythmic encoding remained minimal. The authors conclude that the temporal encoding of a rhythm, whether auditory or visual, relies on the auditory cortex. Auditory capture was probably responsible for the disruption of the visual rhythmic encoding. Since in natural conditions auditory temporal saliency is high and visual capture is almost non-existent, this result is not surprising. However, as demonstrated here, reducing auditory sensitivity with noise could reveal a significant visual capture. The requirement of the auditory cortex to encode visual rhythms could be challenged just by adding the right amount of noise to the irrelevant beep sequences. In conclusion, the findings reported here strongly suggest that auditory preference for timing is not hardwired–a product of evolution. Instead the brain seems to adapt multisensory temporal processing to the context, and accordingly, vision can take over when audition is impaired.

## Conclusion

The motivation behind this project was two-folded. The first goal was to characterize the temporal interactions between discrete auditory and visual events when presented in proximity. The new paradigm allowed quantifying separately the perceptual time shift of each modality within a large range of audiovisual offset. In natural conditions where saliency of both visual and auditory information is regular, the auditory capture of flashes was much stronger than the visual capture of beeps. Combining both visual and auditory time shifts provided an estimation of the perceived distance between the beep and the flash. The outcome changed according to the AV offset from two separated though attracted auditory and visual events, to a single fused bimodal event. Beeps and flashes were perceived as perfectly synchronous within the offset range of –40ms to +40ms, defining the extent of the temporal window of integration. The second goal was to challenge auditory dominance for timing as predicted by the natural modality appropriateness hypothesis, and assess whether the visual capture of beeps can be as efficient as the auditory capture of flashes when both modalities have comparable reliabilities. Auditory sensitivity was impaired embedding the beeps in pink noise and for each participant the SNR for which auditory and visual sensitivity matched was estimated. Noise had a massive impact on the relative contribution of auditory and visual modalities for temporal integration, with the reciprocal sensory attraction shifting the perceived timing of events becoming comparable. This reweighting in the temporal integration demonstrates that auditory preference is not hardwired. The poor sensitivity to visual events observed in natural conditions was in fact responsible for the much weaker attraction of beeps by the flashes.

## Supporting information

S1 Table Experiment 1 & 2 Student’s t-tests and related tests outcome table. The statistical analyses to compare mean time shift and JND for each SOA condition with the no-capture baseline (SOA = 0ms) followed 3 steps: a normality test (Shapiro-Wilk), a variance test (Levene) and a paired Student’s t-test. This table reports the outcome of all these tests together with the effect size (Cohen’s d).
(XLSX)

S2 Table Experiment 4 Student’s t-tests and related tests outcome table. Similar to **S1 Table** for Experiment 1 & 2, this table reports the outcome of all the statistical tests performed for the mean comparison between conditions together with the effect size.
(XLSX)

S1 Fig Adaptive method convergence examples. Two examples of convergence plots of the Bayesian adaptive method taken from experiment 1. The convergence plots of a typical subject for which the estimated mean converged nicely in all conditions (top) and the convergence plots of a subject for which the estimated SD saturates or diverges in some conditions, leading to its exclusion from further analyses (bottom).
(PNG)

S2 Fig Adaptive method test interval distribution. Example of the test interval distribution for each condition. The red dashed line indicates the estimated PSE means at the end of the experiment. Distributions are bimodal and test interval durations close to the estimated PSE were avoided making the task easier for the participants.
(PNG)

S3 Fig Experiment 1 results for the n = 5 subgroup. Time shifts and JNDs obtained in experiment 1 for the subgroup of 5 participants tested in experiment 2. The trends for the full group (n = 10) are very similar than with that subgroup, which legitimizes comparisons between experiment 1 and 2.
(PNG)

S4 Fig Experiment 3 mean PSE results. Average visual PSE (dark gray bar) and auditory PSE according to the signal-to-noise ratio (light gray bars). Error bars indicate the 95% confidence intervals. PSEs were barely different across conditions whichever the modality and noise level.
(PNG)
