# Supplementary figures and images for: Hearing flashes and seeing beeps: Timing audiovisual events
Source: PLoS One. 2017 Feb 16;12(2):e0172028. doi: 10.1371/journal.pone.0172028 (PMC5312923; doi:10.1371/journal.pone.0172028)

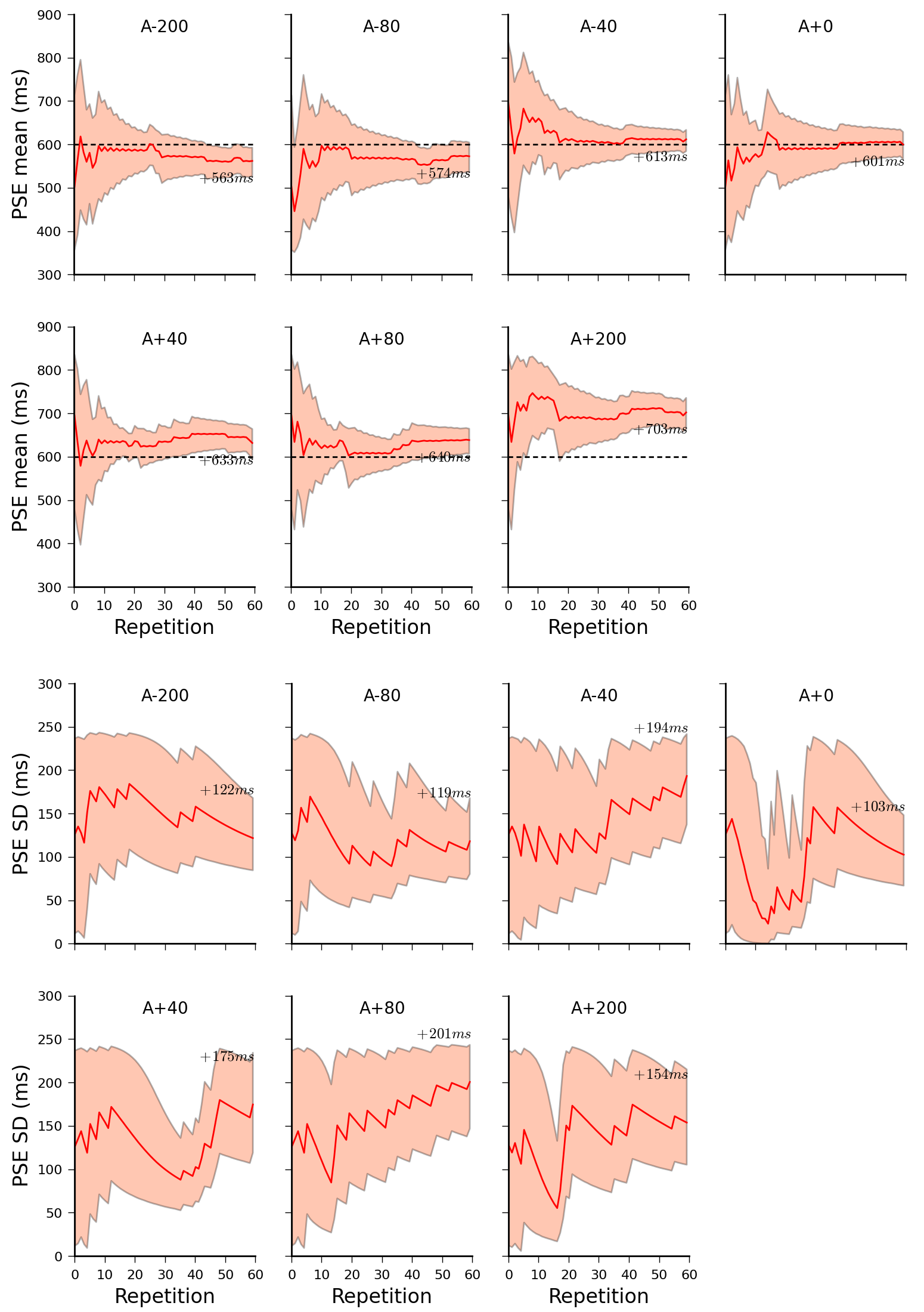

Supplement: S1 Fig — Two examples of convergence plots of the Bayesian adaptive method taken from experiment 1. The convergence plots of a typical subject for which the estimated mean converged nicely in all conditions (top) and the convergence plots of a subject for which the estimated SD saturates or diverges in some conditions, leading to its exclusion from further analyses (bottom). (PNG) [file pone.0172028.s003.png]

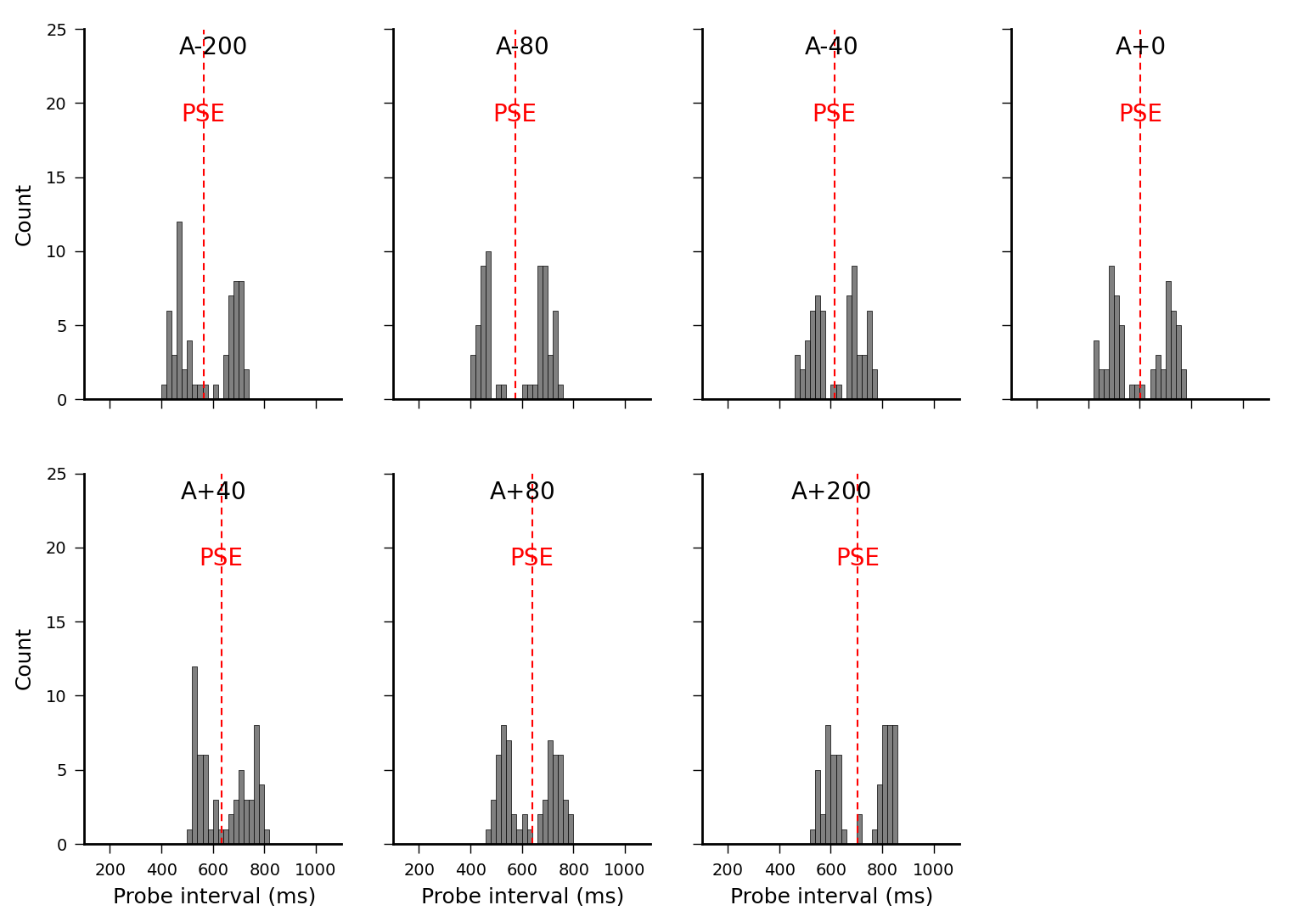

Supplement: S2 Fig — Example of the test interval distribution for each condition. The red dashed line indicates the estimated PSE means at the end of the experiment. Distributions are bimodal and test interval durations close to the estimated PSE were avoided making the task easier for the participants. (PNG) [file pone.0172028.s004.png]

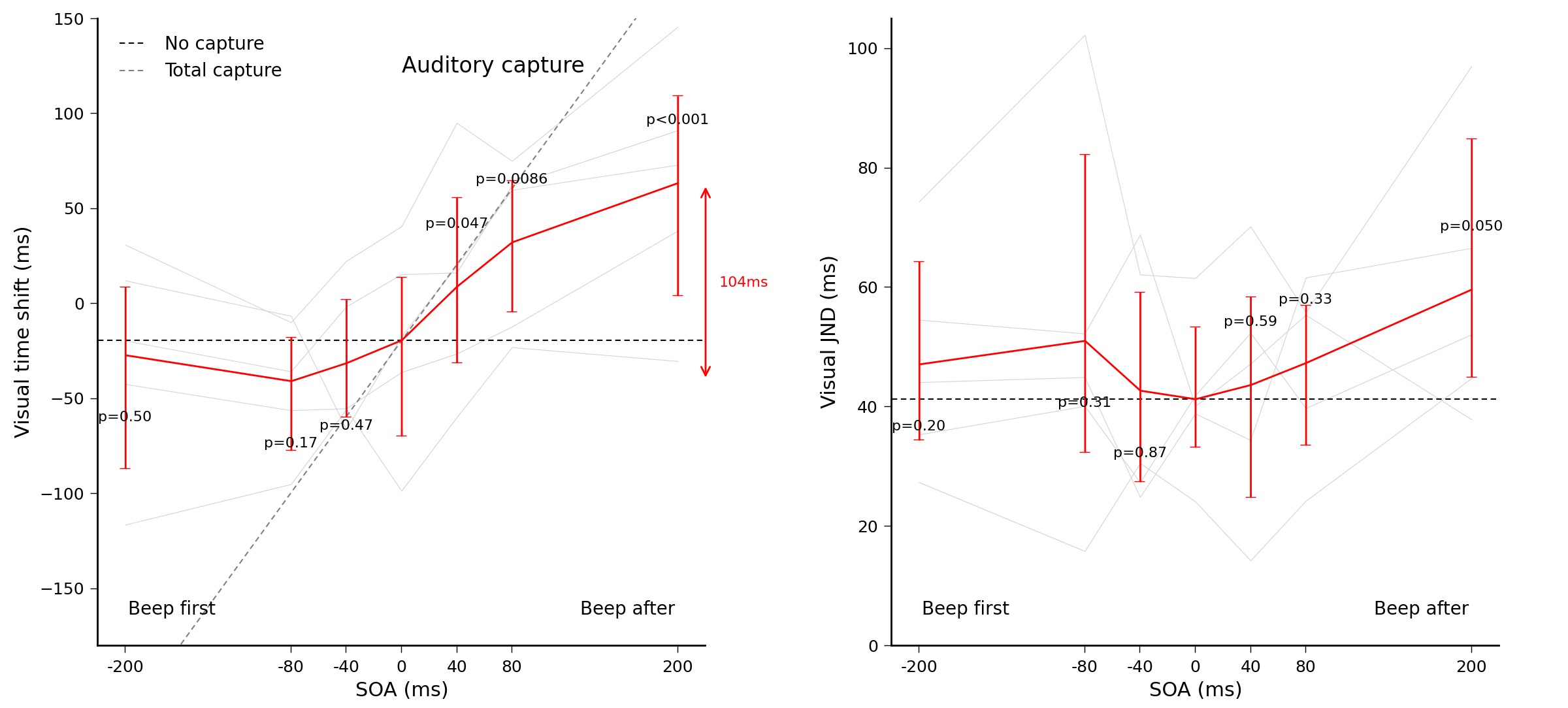

Supplement: S3 Fig — Time shifts and JNDs obtained in experiment 1 for the subgroup of 5 participants tested in experiment 2. The trends for the full group (n = 10) are very similar than with that subgroup, which legitimizes comparisons between experiment 1 and 2. (PNG) [file pone.0172028.s005.png]

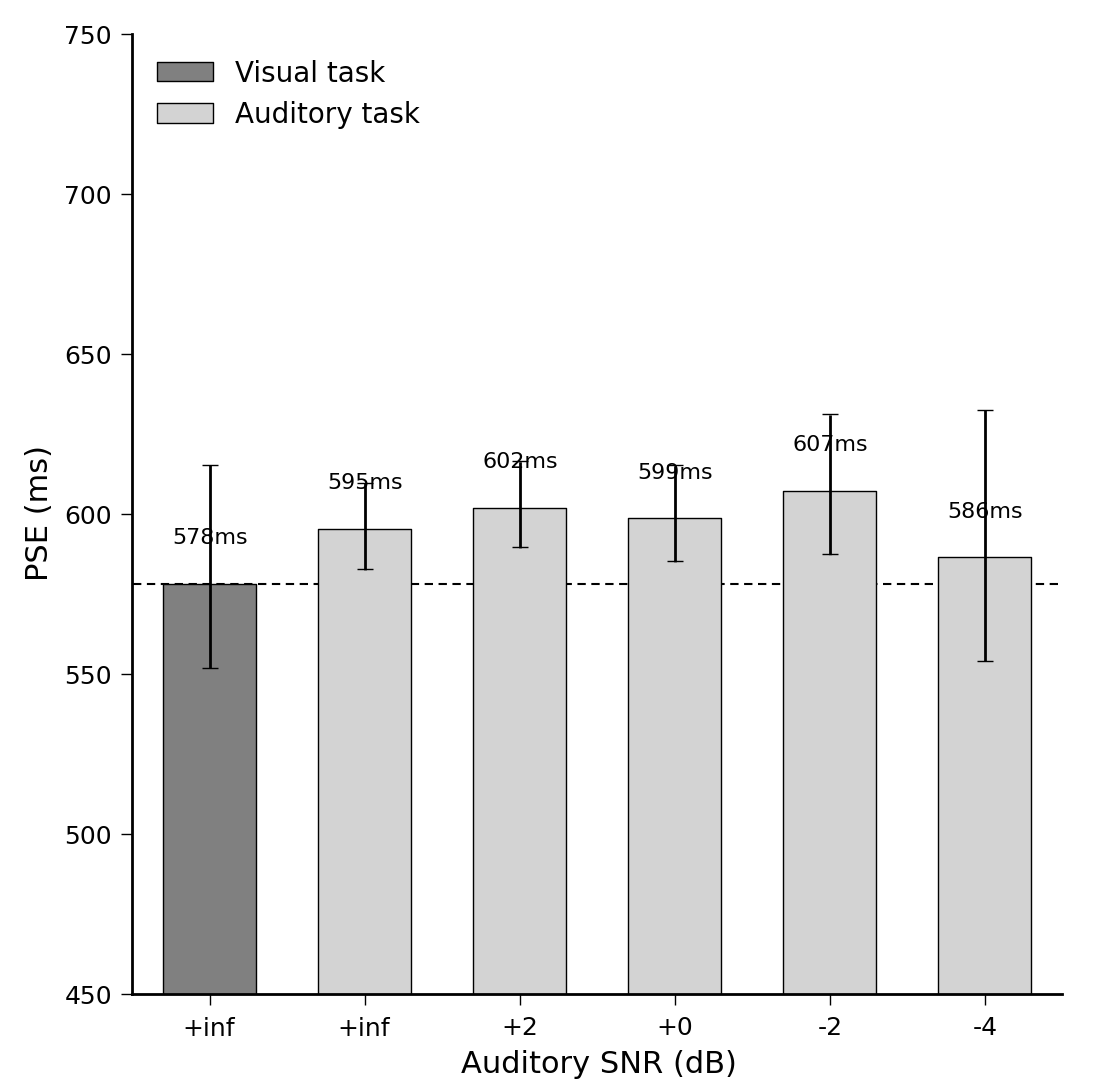

Supplement: S4 Fig — Average visual PSE (dark gray bar) and auditory PSE according to the signal-to-noise ratio (light gray bars). Error bars indicate the 95% confidence intervals. PSEs were barely different across conditions whichever the modality and noise level. (PNG) [file pone.0172028.s006.png]
